# Hyponatremia: A Risk Factor for Early Overt Encephalopathy after Transjugular Intrahepatic Portosystemic Shunt Creation

**DOI:** 10.3390/jcm3020359

**Published:** 2014-04-04

**Authors:** Jonathan Merola, Noami Chaudhary, Meng Qian, Alexander Jow, Katherine Barboza, Hearns Charles, Lewis Teperman, Samuel Sigal

**Affiliations:** 1 Department of Medicine, New York University School of Medicine, New York, NY, 10016, USA; E-Mails: jonathan.merola@nyumc.org (J.M.); noami.chaudhary@nyumc.org (N.C.); alexander.jow@nyumc.org (A.J.); katherine.barboza@nyumc.org (K.B.); 2 Department of Biostatistics, New York University School of Medicine, New York, NY 10016, USA; E-Mail: meng.qian@nyumc.org; 3 Department of Radiology, New York University School of Medicine, New York, NY 10016, USA; E-Mail: hearns.charles@nyumc.org; 4 Department of Surgery, New York University School of Medicine, New York, NY 10016, USA; E-Mail: lewis.teperman@nyumc.org

**Keywords:** hyponatremia, hepatic encephalopathy, TIPS

## Abstract

Hepatic encephalopathy (HE) is a frequent complication in cirrhotic patients undergoing transjugular intrahepatic portosystemic shunt (TIPS). Hyponatremia (HN) is a known contributing risk factor for the development of HE. Predictive factors, especially the effect of HN, for the development of overt HE within one week of TIPS placement were assessed. A single-center, retrospective chart review of 71 patients with cirrhosis who underwent TIPS creation from 2006–2011 for non-variceal bleeding indications was conducted. Baseline clinical and laboratory characteristics were collected. Factors associated with overt HE within one week were identified, and a multivariate model was constructed. Seventy one patients who underwent 81 TIPS procedures were evaluated. Fifteen patients developed overt HE within one week. Factors predictive of overt HE within one week included pre-TIPS Na, total bilirubin and Model for End-stage Liver Disease (MELD)-Na. The odds ratio for developing HE with pre-TIPS Na <135 mEq/L was 8.6. Among patients with pre-TIPS Na <125 mEq/L, 125–129.9 mEq/L, 130–134.9 mEq/L and ≥135 mEq/L, the incidence of HE within one week was 37.5%, 25%, 25% and 3.4%, respectively. Lower pre-TIPS Na, higher total bilirubin and higher MELD-Na values were associated with the development of overt HE post-TIPS within one week. TIPS in hyponatremic patients should be undertaken with caution.

## 1. Introduction

Hepatic encephalopathy (HE) is a frequent complication of liver disease affecting 50%–70% of patients with cirrhosis [1]. Defined by the West Haven criteria, HE represents a range of neuropsychiatric abnormalities, from mildly abnormal psychometric testing, to severe neurologic dysfunction, in which asterixis, impaired cognition, lethargy and coma are present [2]. These latter grades have been termed overt HE.

Multiple mechanisms have been proposed for the development of HE [3]. The most important contributor is the presence of an elevated serum ammonia level due to impaired hepatic function and portosystemic shunting. Hyperammonemia leads to HE by altering the osmotic gradient within astrocytes, changing intracellular glutamine-glutamate metabolism, decreasing cerebral blood flow and cerebral oxygen metabolism [3,4,5]. Elevated serum ammonia leads to the conversion and accumulation of glutamine from glutamate. Glutamine, an active organic osmolyte, leads to astrocytic edema and impairs neurotransmission [6,7].

Dilutional hyponatremia (HN), due to the non-osmotic release of arginine vasopressin (AVP) into the bloodstream, occurs in 30% of cirrhotics and is associated with severe portal hypertension. In non-cirrhotic patients, HN is associated with a spectrum of neurologic findings that closely mirrors HE. Clinically, HN exacerbates HE in cirrhosis, as it worsens ammonia-induced brain edema by depleting organic osmolytes and impairing astrocyte osmotic compensatory ability [8]. HN is directly correlated with the incidence of HE and predictive of its subsequent development [8,9].

The transjugular intrahepatic portosystemic shunt (TIPS) involves a procedure in which an expandable metal stent is placed within the liver to provide a communicating pathway from the portal to the hepatic vein used to treat complications of portal hypertension, such as variceal bleeding and refractory ascites [10,11,12]. A major complication of TIPS is the development of encephalopathy [12,13,14]. Risk factors for the development of HE after TIPS include older age, a history of HE, a low portal pressure gradient after TIPS, higher Child-Pugh class, high creatinine, low albumin levels and low serum sodium (Na) [13,14,15]. Given the shared pathophysiologic process by which HN and hyperammonemic states lead to neurologic dysfunction via astrocytic edema, we speculated that the presence of HN at the time of TIPS would increase the incidence of overt encephalopathy immediately after TIPS. In this study, we assess risk factors, specifically the effect of HN, for the development of overt HE within one week in patients undergoing TIPS.

## 2. Experimental Methods

### 2.1. Patient Selection

The Vascular and Interventional Radiology database of patients undergoing the placement of a TIPS between January 2006 and December 2011, at the New York University Langone Medical Center was retrospectively reviewed for patients undergoing the procedure electively for non-variceal bleeding indications. Patients on renal-replacement therapy, requiring mechanical ventilation, or post-liver transplant were excluded, as these factors are known to potentiate the development of HE. We deliberately excluded variceal bleeding in this study to limit factors outside the TIPS creation influencing the development of early overt hepatic encephalopathy. Variceal bleeding in an emergent setting may confound the symptoms of overt encephalopathy. Indications for TIPS in our cohort in large part include refractory ascites and partial portal vein thrombosis, for the maintenance of the listing status for transplantation. In the latter case, patients may have had undergone TIPS in the setting of mild HE after closely weighing risks and benefits. All patients underwent TIPS following consent by the same team of interventional radiologists. The study protocol was approved by the New York University School of Medicine Institutional Review Board (IRB).

### 2.2. Patient Characteristics

Demographic, clinical and biochemical data were collected from medical records using data collection forms, including the history of overt HE, HE therapy, ascites, indications for TIPS creation, Child-Pugh (CP) score, the Model for End-stage Liver Disease (MELD) score and MELD-Na (Table 1). The presence of cirrhosis was based on clinical data (*i.e*., a history of liver disease, the results of imaging, laboratory studies and liver biopsy). A history of overt HE required the documentation of an altered level of consciousness, confusion, disorientation or coma. The Na level and the average of serum Na concentration for the 3 days prior to TIPS insertion (pre-TIPS Na) were recorded. Daily serum sodium for 3 days prior to TIPS was not always available, as some patients in this cohort had ambulatory pre-procedural testing. In those cases, the average of Na levels drawn within 3 days of the procedure were used. A normal Na level was defined as ≥135 mEq/L. Patients with an Na level 130 to <135 mEq/L were considered as having mild HN, 125 to <130 mEq/L as moderate HN and <125 mEq/L as severe HN.

### 2.3. Post-TIPS Outcomes

Patient records were assessed for the development of overt HE as defined by the documentation of an altered level of consciousness, somnolence, confusion, disorientation or coma. Early overt HE (EOE) was defined as its development within the first week after TIPS. Patients did not undergo psychometric testing. Prophylactic use of Lactulose and/or Rifaximin following TIPS was recorded. The length of stay after TIPS and in-hospital mortality were also assessed.

**Table 1:** Demographics and clinical characteristics of patients undergoing transjugular intrahepatic portosystemic shunt (TIPS) insertion for non-variceal bleeding indications. Patients managed with medical therapy were categorized as West-Haven Grade 2. EOE, early overt HE; HE, hepatic encephalopathy; MELD, Model for End-stage Liver Disease; HCV, hepatitis C virus; HBV, hepatitis B virus; AST, aspartate aminotransferase; ALT, alanine aminotransferase; INR, international normalized ratio.

	Overall	EOE	No EOE	*p*
	(*N* = 81)	(*N* = 15)	(*N* = 66)	
**Demographics **				
Male	58 (72%)	12 (80%)	46 (70%)	0.42
Female	23 (28%)	3 (20%)	20 (30%)	0.42
Mean age ± SD	57 ± 8	60 ± 6	57 ± 8	0.10
Etiology (*N*) *				
HCV/HBV	50 (62%)	10 (67%)	40 (61%)	0.67
Alcohol	16 (20%)	4 (27%)	12 (18%)	0.45
Other	15 (19%)	1 (7%)	14 (21%)	0.19
**Clinical Characteristics**				
Refractory Ascites	69 (85%)	15 (100%)	54 (81%)	0.07
TIPS Stenosis	3 (4%)	0 (0%)	3 (5%)	0.40
Hydrothorax	14 (17%)	3 (20%)	11 (17%)	0.76
Portal Vein Thrombosis	7 (9%)	1 (7%)	6 (9%)	0.76
History of Overt HE	24 (30%)	2 (13%)	22 (33%)	0.13
Pre-TIPS HE Prophylaxis	55 (68%)	11 (73%)	44 (67%)	0.62
**Laboratory Parameters (mean ± SD)**				
Serum [Na^+^] (Normal = 133–146 mEq/L) **	132.7 ± 5.2	130.4 ± 4.0	133.3 ± 5.3	0.02
Serum pre-TIPS [Na^+^] (133–146 mEq/L) **	132.6 ± 5.3	129.6 ± 5.0	133.3 ± 5.1	0.01
Creatinine (0.6–1.3 mg/dL)	1.3 ± 0.9	1.2 ± 0.6	1.3 ± 1.0	0.61
Albumin (3.5–5.0 g/dL)	2.6 ± 0.7	2.8 ± 0.7	2.5 ± 0.7	0.13
AST (5–40 IU/L)	80 ± 79	104 ± 159	75 ± 45	0.48
ALT (7–56 IU/L)	55 ± 49	64 ± 83	53 ± 38	0.62
INR (0.8–1.2)	1.6 ± 0.4	1.8 ± 0.5	1.6 ± 0.3	0.14
Alkaline Phosphatase (40–140 IU/L)	135 ± 65	154 ± 97	131 ± 55	0.38
Total Bilirubin (0.1–1.2 mg/dL)	3.1 ± 2.6	4.6 ± 4.5	2.7 ± 1.8	0.11
**MELD and Child-Pugh Scores**				
MELD	17 ± 4	19 ± 5	17 ± 4	0.15
MELD-Na	21 ± 5	24 ± 5	20 ± 5	0.01
Child-Pugh Score	11 ± 2	11 ± 2	10 ± 2	0.08

* Note that several patients had multiple factors contributing to liver cirrhosis and indications for TIPS; ** Pre-TIPS Na indicates the average of serum Na concentration for the three days prior to TIPS insertion. Serum [Na^+^] denotes the sodium level on the day TIPS was performed.

### 2.4. Statistical Analysis

Descriptive statistics were used to summarize patient characteristics. The univariate logistic regression model was used to assess the factors associated with the development of EOE. Pearson correlation analysis was conducted on identified risk factors. All potential risk factors were eligible to be included in the multivariate logistic regression model, where variables were selected by minimizing Akaike’s information criterion. Odds ratios (OR) were calculated to determine the relative risk of developing EOE for each of the predictive variables. The area under the receiver operating characteristic curve (AUC) was calculated as an indication of the discriminative power of the logistic predictive models. Statistical significance of tests was claimed when *p* < 0.05. Statistical analyses were conducted using SAS (Version 9.2, Cary, NC: SAS Institute Inc., 2009).

## 3. Results and Discussion

### 3.1. Patient Characteristics

Seventy-one subjects undergoing 81 successful TIPS creations met the inclusion criteria. Patient characteristics are presented in Table 1. Indications for TIPS included refractory ascites (*N* = 69), hydrothorax (*N* = 14), portal vein thrombosis (*N* = 7) and TIPS stenosis (*N* = 3). Twenty-five patients (35%) had no evidence or active treatment for HE. Forty-six (65%) were receiving Lactulose and/or Rifaximin prior to TIPS insertion, among which, 23 had a documented clinical history of overt HE, while others were started on therapy prophylactically by their physician, with clinically evident signs of mild HE prior to TIPS (Table 1). No patients had overt HE at the time of TIPS insertion. Among the 25 patients who were previously not on treatment for HE, 21 (84%) received Lactulose and Rifaximin immediately following TIPS. Normal Na levels at the time of TIPS were observed in 41% of patients (*N* = 33), and HN was present in 59% (*N* = 48) (mild, 34%; moderate, 16%; severe, 9%). Pre-TIPS Na levels were normal in 36% (*N* = 29) (mild, 34%; moderate, 20%; severe, 10%). The mean Child-Pugh (CP) score was 11 ± 2 (range: 7–14) (CP Class A, zero; CP Class B, 27; CP Class C, 54). The mean MELD score was 17 ± 4 (range: 6–29), and the mean MELD-Na was 21 ± 5 (range: 6–33).

### 3.2. Post-TIPS Outcomes

#### 3.2.1. Development of Early Overt Encephalopathy (EOE)

**Figure 1:**
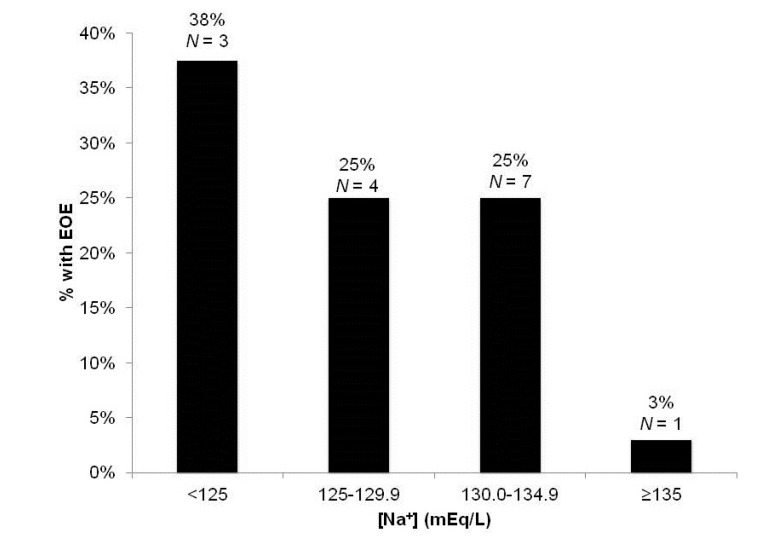
Percentage of patients developing early overt encephalopathy (EOE) by pre-TIPS Na (mEq/L).

**Figure 2:**
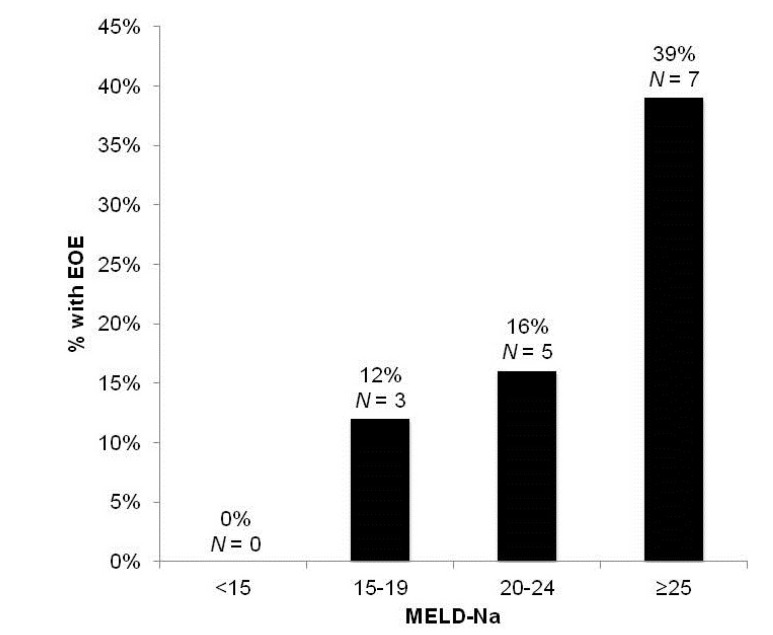
Percentage of patients developing early overt encephalopathy (EOE) by MELD-Na group.

EOE occurred in 15 of 81 total TIPS creations (18.5%). The relationship between the various clinical variables and EOE are presented in Table 2. Significant factors included pre-TIPS Na, MELD-Na scores and total bilirubin (*p* < 0.05). Fourteen of the 15 patients (93.3%) who developed EOE had a pre-TIPS Na <135 mEq/L. Among patients with pre-TIPS Na ≥135 mEq/L, 130 to <135 mEq/L, 125 to <130 mEq/L and Na <125 mEq/L and the incidence of EOE was 3.4%, 25.0%, 25.0% and 37.5%, respectively (Figure 1). The incidence of EOE also increased with increasing MELD-Na (<15, 0%; 15–19, 12%; 20–24, 16%; ≥25, 39%) (Figure 2). Age, albumin, CP scores, history of overt HE and creatinine levels did not significantly differ between patients with and without EOE (*p* > 0.05).

**Table 2:** Factors associated with early overt encephalopathy and length of stay as determined by Wald (χ^2^) univariate regression analysis. Pre-TIPS Na indicates the average of serum Na concentration for the three days prior to TIPS insertion; SBP, spontaneous bacterial peritonitis; PVT, portal vein thrombosis; HCC, hepatocellular carcinoma; HE, hepatic encephalopathy. * *p* < 0.01; LOS, length of stay.

Variable	EOE (*p*)	LOS (*p*)
Age	0.15	0.23
Pre-TIPS [Na^+^]	0.03 *	<0.01 *
Creatinine	0.83	0.19
Albumin	0.12	0.04 *
AST	0.25	0.25
ALT	0.43	0.17
INR	0.13	0.01 *
Total Bilirubin	0.02 *	<0.01 *
MELD	0.10	<0.01 *
MELD-Na	<0.01 *	<0.01 *
Childs-Pugh Score	0.19	<0.01 *
Ascites	0.24	0.31
Portal Vein Thrombosis	0.96	0.61
History of HE	0.90	0.78
Diabetes mellitus (DM)	0.55	0.81
Insulin-Dependent DM	0.42	0.05 *

Paired correlation analysis conducted on the three predictor variables revealed that they were strongly correlated with each other (*p* < 0.001 in all cases). To reduce the co-linearity between the predictors, continuous variables were converted into categorical variables, and combinations of categorical and continuous variables were utilized to build a multivariate model. A model selection procedure was used to identify total bilirubin (continuous variable) and a pre-TIPS Na with a cutoff value of 135 mEq/L (categorical value) as the best-fit model with an AUC = 0.712 (Table 3). EOE was therefore best predicted by the total bilirubin concentration and a mean serum Na concentration for the three days prior to TIPS insertion (pre-TIPS Na) of <135 mEq/L.

Table 3(**A**) In the best fit multivariate model, pre-TIPS Na was divided into ≥135 and a <135 (mEq/dL) categorical variable, and total bilirubin was included as a continuous variable; (**B**) Odds-ratio estimates.

**Table:** **(A)**

Parameter	Odds-Ratio	Standard Error	Wald (χ^2^)
Intercept	−1.76	0.52	11.38
Pre-TIPS Na	−2.16	1.07	4.03
Total Bilirubin	0.21	0.11	3.76

**Table:** **(B)**

Factor	Odds Ratio	95% Confidence Interval
Pre-TIPS Na ≥135 (mEq/L)	0.52	1.05–71.43
Total Bilirubin	1.07	1.00–1.51

#### 3.2.2. Length of Stay after TIPS Insertion

The average length of stay (LOS) of patients with and without EOE was 15.4 ± 19.8 days and 4.4 ± 5.1 days, respectively (*p* < 0.001). Correlations of the various clinical parameters with post-TIPS LOS are presented in Table 3. Factors closely associated with LOS on univariate analysis included low pre-TIPS Na and high total bilirubin, MELD, MELD-Na and CP scores (*p* < 0.05).

#### 3.2.3. In-Hospital Mortality

Four (4.9%) of the 81 patients undergoing TIPS were associated with hospital mortality, including three patients (75%) with EOE. Prognostic variables predicting death in the univariate analysis were high total bilirubin, MELD, MELD-Na and CP scores.

### 3.3. Discussion

A transjugular intrahepatic portosystemic shunt (TIPS) is an effective therapy for complications of portal hypertension and is frequently employed as a temporizing measure for patients awaiting liver transplantation. The most common complication of TIPS creation is the development of HE, occurring in 30%–60% of patients within one year [13]. The development of overt HE following TIPS creation is associated with significant morbidity and is a predictor of mortality [14,16,17,18]. As a result, the identification of pre-procedural risk factors that are potentially modifiable is of paramount importance.

In this retrospective study, we identify the factors predictive of EOE within one week of TIPS. The short post-procedural timeframe distinguishes acute risk factors for post-TIPS encephalopathy from variables associated with the chronic progression of cirrhosis. Among the 81 cases undergoing TIPS for non-variceal bleeding indications, fifteen developed EOE. The most significant predictors included pre-TIPS Na and total bilirubin levels. Of those who developed EOE, 93.3% had a pre-TIPS Na <135 mEq/L. In addition, there was an inverse relationship between the severity of HN and the risk of EOE. EOE was observed in 38%, 25%, 25% and 3% of patients with pre-TIPS Na levels of <125, 125–129.9, 130–134.9 and ≥135 mEq/L, respectively. Furthermore, low pre-TIPS Na and high total bilirubin levels were important predictors of post-procedural LOS, independent of MELD score. Although the small number of deaths precluded a meaningful analysis for the factors predictive of hospital mortality, three of the four patients who died had HN.

Hyperammonemia and resultant astrocytic swelling are central events in the proposed models for HE pathogenesis. Because of the presence of glutamine synthetase within astrocytes, elevated ammonia levels lead to increased intracellular concentrations of glutamine, resulting in increased intracellular osmolality [19]. The increased osmolality leads to the passage of extracellular fluid into the intracellular compartment, intracellular depletion of compensatory organic osmolytes, such as myo-inositol, and astrocytic swelling and dysfunction [20,21]. The low-grade cerebral edema impairs neurotransmission and clinically manifests as HE [3]. The model of astrocyte edema leading to HE is supported by magnetic resonance studies in cirrhotic patients that demonstrate increased glutamine/glutamate signal and myo-inositol depletion, consistent with partially compensated glial edema [8,22,23,24]. Moreover, cirrhotics with lower brain myo-inositol have a significantly higher probability of developing overt HE compared with those with higher myo-inositol levels at three months [8].

Hyponatremia (HN) is the most commonly encountered electrolyte abnormality in hospitalized patients and associated with a range of neurologic manifestations, due to increased brain edema associated with myo-inositol depletion. Symptoms closely parallel those of HE and range from mild disturbances in gait and attention to depressed sensorium and seizures in severe cases [25,26,27]. Animal studies have demonstrated that severe neurological symptoms develop in hyponatremic individuals when additional stressors, such as hypoxia, are concurrently present [28].

Dilutional HN commonly develops in patients with advanced cirrhosis and portal hypertension as a result of the non-osmotic release of vasopressin, due to decreased effective blood volume from splanchnic vasodilation [29,30,31]. It is especially common in patients with refractory ascites in whom TIPS may be indicated. In these patients, diuretic therapy exacerbates the process by inducing intravascular volume depletion and non-osmotic release of AVP [32]. In addition, diuretics impair the reclamation of sodium and chloride, leaving increased free water reabsorption, due to the increased release of AVP being unopposed [32].

HN is closely associated with the development of HE in cirrhosis, and a higher incidence of HE is observed among patients with HN [9]. In a prospective study of 997 patients, HN (serum sodium <135 mEq/L) was associated with both refractory ascites and a greater frequency of HE. Although patients with serum sodium <130 mEq/L had the greatest frequency of these complications, the frequency was also increased in patients with a mild reduction in serum sodium levels (131–135 mEq/L) [9]. Finally, a strong inverse relationship between serum sodium and the incidence of HE was observed [9]. In a prospective study of 61 patients with cirrhosis followed over a one-year period, HN (serum sodium <130 mEq/L) was the strongest independent predictor for the development of overt HE [8].

HN exacerbates the effects of increased ammonia levels and increases the risk of HE in cirrhotic patients by further depleting the cerebral concentration of organic osmolytes, especially myo-inositol, and the osmoregulatory capacity of cerebral astrocytes [8,20,33,34]. Patients with lower brain myo-inositol levels have a higher probability of developing overt HE compared with those with higher myo-inositol (83% *vs*. 31% at three months) [6]. Among outpatient cirrhotic patients, HN has been shown to have a detrimental impact on psychomotor abilities in individuals with HE [35]. It has been proposed that HN increases the severity of astrocytic edema by imposing an additional osmoregulatory stressor in the setting of pre-existing astrocytic edema resultant from hyperammonemia [8]. Quantitative electroencephalography has been used as a tool to measure neurotransmission impairments in cirrhotics and is predictive of the occurrence of overt HE. A recent study demonstrated that high ammonia and low sodium levels were the strongest predictors of EEG alterations in cirrhotic patients [36]. While HN may not be sufficient to trigger HE alone, it is a “second hit”, which further precipitates the progression of HE [8,33].

The compensated osmotic balance resultant from chronic hepatic dysfunction may be offset following TIPS creation in which a large ammonia load is acutely delivered to cerebral astrocytes. The resultant edema may rarely be accompanied by a rise in intracranial pressure, resulting in seizures and death [37]. More commonly, worsening or new-onset overt HE develops after TIPS creation, occurring in 30%–60% over one year [13,38,39,40]. Most cases of post-TIPS HE occur during the early post-TIPS period, especially within one to three months of TIPS [40,41]. In a prospective study of 87 patients undergoing TIPS, HE occurred with an incidence of 50.5% at one year, with 17% experiencing HE during the same hospitalization of TIPS placement and 76% of episodes occurring within three months [42]. Among 55 patients followed prospectively for HE after TIPS, the cumulative rate of HE in the first three months after TIPS increased from 23.6% (prior to TIPS creation) to 50.9%. *De novo* HE developed in 30.9% of patients, and the proportion of overt HE increased from 10% to 22.5% by one month following TIPS [38]. Finally, in a study of 77 patients undergoing TIPS, the overall incidence of clinically significant new or worsened encephalopathy after TIPS was 23%, with a mean time to the onset of encephalopathy of 26 days. Among those who developed HE, 90% did so within 45 days of TIPS [38].

Numerous studies have attempted to identify risk factors for the development of post-TIPS HE over time periods ranging from one month to one year [13,40,41,42,43]. A recent review found that increased age, a history of HE and a higher Child-Pugh class/score were the most robust predictors for post-TIPS HE [13]. Other studies have demonstrated high creatinine levels, low albumin levels and low serum Na to be independent predictive factors for the occurrence of HE after TIPS [13,14]. Among 87 patients followed for a mean of 30.9 months, risk factors that have been identified for post-TIPS HE included older age and ascites as an indication [39]. The long follow-up period in these studies, however, and the natural history of end-stage liver disease make it difficult to distinguish whether the onset of post-TIPS HE is associated with the rapid progression of liver disease or with the identified pre-procedural risk factors.

Animal models of cirrhosis demonstrate that chronic hyponatremia worsens brain edema following portacaval anastomosis [7]. In an analysis of 70 patients with cirrhosis and refractory ascites comparing TIPS with large-volume paracentesis, hyponatremia, serum bilirubin and serum creatinine were independently associated with the development of HE [43]. Riggio *et al*. also reported HN as a risk factor for post-TIPS HE, but the relationship of the timing of the HE episodes with HN was not provided [13]. Our study is the first to evaluate risk factors for overt HE during the early post-procedure period. In this study, we speculated that HN would play an important role in the development of EOE, because the procedure corrects the ineffective intravascular volume depletion that is the cause for HN. Of note, Riggio *et al*. did not find a relationship between HN and refractory HE. Because HN was most likely corrected after the procedure, one would not expect it to be related to a long-term condition, such as refractory HE. In contrast to prior studies, older age, a history of HE, a high Child-Pugh score, low albumin or high creatinine were not associated with post-TIPS HE. Possible explanations for this discrepancy include: (i) the small number of HE events in our cohort; (ii) these risk factors predisposing to the onset of HE outside the one-week follow-up; and (iii) these risk factors being harbingers of progressing liver disease and not the development of acute, post-procedure HE.

Our findings are subject to several limitations. Our study was a retrospective chart review with a relatively small number of subjects. Moreover, the development of HE was based on the clinical documentation of HE in the medical record and not prospective psychometric testing. Despite these limitations, we demonstrate that pre-TIPS Na is a predictor of EOE following TIPS creation and, of increasing importance in an era of increasing medical expenditures, of increased LOS. In addition to HE, HN has been found to be an independent predictor of mortality in patients undergoing TIPS. A study of 68 patients undergoing TIPS for variceal hemorrhage revealed that sodium <135 mEq/L was a significant predictor of both 30-day (30% *vs*. 5%) and one-year (59% *vs*. 14%) mortality [14].

## 4. Conclusions

TIPS insertion in patients with HN should be undertaken with caution. Future studies are required to determine whether the correction of pre-TIPS HN will decrease the risk of EOE.

## References

[1] Riordan S.M., Williams R. (1997). Treatment of hepatic encephalopathy. N. Engl. J. Med..

[2] Atterbury C.E., Maddrey W.C., Conn H.O. (1978). Neomycin-sorbitol and lactulose in the treatment of acute portal-systemic encephalopathy. A controlled, double-blind clinical trial. Am. J. Dig. Dis..

[3] Blei A.T. (2005). The pathophysiology of brain edema in acute liver failure. Neurochem. Int..

[4] Dam G., Keiding S., Munk O.L., Ott P., Vilstrup H., Bak L.K., Waagepetersen H.S., Schousboe A., Sørensen M. (2013). Hepatic encephalopathy is associated with decreased cerebral oxygen metabolism and blood flow not increased ammonia uptake. Hepatology.

[5] Iversen P., Sørensen M., Bak L.K., Waagepetersen H.S., Vafaee M.S., Borghammer P., Mouridsen K., Jensen S.B., Vilstrup H., Schousboe A. (2009). Low cerebral oxygen consumption and blood flow in patients with cirrhosis and an acute episode of hepatic encephalopathy. Gastroenterology.

[6] Blei A.T., Olafsson S., Therrien G., Butterworth R.F. (1994). Ammonia-induced brain edema and intracranial hypertension in rats after portacaval anastomosis. Hepatology.

[7] Cordoba J., Gottstein J., Blei A.T. (1998). Hyponatremia exacerbates ammonia-induced brain edema in rats after portacaval anastomosis. J. Hepatol..

[8] Guevara M., Baccaro M.E., Torre A., Gomez-Anson B., Rios J., Torres F., Rami L., Monte-Tubio G.C., Martin-Llahi M., Arroyo V. (2009). Hyponatremia is a risk factor of hepatic encephalopathy in patients with cirrhosis: A prospective study with time-dependent analysis. Am. J. Gastroenterol..

[9] Angeli P., Wong F., Watson H., Gines P. (2006). Hyponatremia in cirrhosis: Results of a patient population survey. Hepatology.

[10] Rossle M., Haag K., Ochs A., Sellinge M., Noldge G., Perarnau J.M., Berger E., Blum U., Gabelmann A., Hauenstein K. (1994). The transjugular intrahepatic portosystemic stent-shunt procedure for variceal haemorrhage. N. Engl. J. Med..

[11] LaBerge J.M., Somberg K.A., Lake J.R., Gordon R.L., Kerlan R.K., Ascher N.L., Roberts J.P., Simor M.M., Doherty C.A., Hahn J. (1995). Two-year outcome following transjugular intrahepatic portosystemic stent-shunt for variceal bleeding: Results in 90 patients. Gastroenterology.

[12] Russo M.W., Sood A., Jacobson I.M., Brown R.S. (2003). Transjugular intrahepatic portosystemic shunt for refractory ascites: An analysis of the literature on efficacy, morbidity, and mortality. Am. J. Gastroenterol..

[13] Riggio O., Angeloni S., Salvatori F.M., de Santis A., Cerini F., Farcomeni A., Attili A.F., Merli M. (2008). Incidence, natural history and risk factors of hepatic encephalopathy after transjugular intrahepatic portosystemic with polytetrafluoroethylene-covered stent grafts. Am. J. Gastroenterol..

[14] Jalan R., Elton R.A., Redhead D.N., Finlayson N.D., Hayes P.C. (1995). Analysis of prognostic variables in the prediction of mortality, shunt failure, varicealrebleeding and encephalopathy following the transjugular intrahepatic portosystemic stent-shunt for variceal hemorrhage. J. Hepatol..

[15] Bai M., Qi X., Yang Z., Fan D., Han G. (2011). Predictors of hepatic encephalopathy after transjugular intrahepatic portosystemic shunt in cirrhotic patients: A systematic review. J. Gastroenterol. Hepatol..

[16] Zuckerman D.A., Darcy M.D., Bocchini T.P., Hildebolt C.F. (1997). Encephalopathy after transjugular intrahepatic portosystemic shunting: Analysis of incidence and potential risk factors. Am. J. Roentgenol..

[17] Poordad F.F. (2007). The burden of hepatic encephalopathy. Aliment. Pharmacol. Ther..

[18] Leevy C.B., Phillips J.A. (2007). Hospitalizations during the use of rifaximin *vs*. lactulose for the treatment of hepatic encephalopathy. Dig. Dis. Sci..

[19] Swain M., Butterworth R.F., Blei A.T. (1992). Ammonia and related amino acids in the pathogenesis of brain edema in acute ischemic liver failure in rats. Hepatology.

[20] Skowrońska M., Albrecht J. (2013). Oxidative and nitrosative stress in ammonia neurotoxicity. Neurochem. Int..

[21] Häussinger D., Görg B. (2010). Interaction of oxidative stress, astrocyte swelling and cerebral ammonia toxicity. Curr. Opin. Clin. Nutr. Metab. Care.

[22] Cordoba J., Alonso J., Rovira A., Jacas C., Sanpedro F., Castells L., Vargas V., Margarit C., Kulisewsky J., Esteban R. (2001). The development of low-grade cerebral edema in cirrhosis is supported by the evaluation of ^1^H-magnetic resonance abnormalities after liver transplantation. J. Hepatol..

[23] Lodi R., Tonon C., Stracciari A., Weiger M., Camaggi V., Iotti S., Donati G., Guarino M., Bolondi L., Barbiroli B. (2004). Diffusion MRI showes increase water apparent diffucsion coefficient in the brains of cirrhotics. Neurology.

[24] Haussinger D. (2006). Low grade cerebeal edema and the pathogenesis of hepatic encephalopathy in cirrhosis. Hepatology.

[25] Decaux G. (2006). Is asymptomatic hyponatremia really asymptomatic?. Am. J. Med..

[26] Schrier R.W. (2010). Does “asymptomatic hyponatremia” exist?. Nat. Rev. Nephrol..

[27] Kokko J.P. (2006). Symptomatic hyponatremia with hypoxia is a medical emergency. Kidney Int..

[28] Ayus J.C., Armstrong D., Arieff A.I. (2006). Hyponatremia with hypoxia: Effects on brain adaptation, perfusion and histology in rodents. Kidney Int..

[29] Gines P., Berl T., Bernardi M., Bichet D.G., Harmon G., Jimenez W., Liard J.F., Martin P.Y., Schrier R.W. (1998). Hyponatremia in cirrhosis: From pathogenesis to treatment. Hepatology.

[30] Bichet D., Szatalowicz V., Chaimovitz C., Schrier R.W. (1982). Role of vasopressin in abnormal water excretion in cirrhotic patients. Ann. Intern. Med..

[31] Epstein M. (1985). Derangement of renal water handling in liver disease. Gastroenterology.

[32] Liamis G., Milionis H., Elisf M. (2008). A review of drug-induced hyponatremia. Am. J. Kidney Dis..

[33] Frederick R.T. (2011). Current concepts in the pathophysiology and management of hepatic encephalopathy. J. Gastroenterol. Hepatol..

[34] Restuccia T., Gomez-Anson B., Guevara M., Alessandria C., Torre A., Alayrach M.E., Terra C., Martin M., Castellvi M., Rami L. (2004). Effects of dilutionalhyponatremia on brain organic osmolytes and water content in patients with cirrhosis. Hepatology.

[35] Ahluwalia V., Wade J.B., Thacker L., Kraft K.A., Sterling R.K., Stravitz R.T., Fuchs M., Bouneva I., Puri P., Luketic V. (2013). Differential impact of hyponatremia and hepatic encephalopathy on health-related quality of life and brain metabolite abnormalities in cirrhosis. J. Hepatol..

[36] Amodio P., Del Piccolo F., Petteno E., Mapelli D., Angeli P., Iemmolo R., Muraca M., Musto C., Gerunda G., Rizzo C. (2001). Prevalence and prognostic value of quantified electroencephalogram (EEG) alterations in cirrhotic patients. J. Hepatol..

[37] Jalan R., Dabos K., Redhead D.N., Lee A., Hayes P.C. (1997). Elevation of intracranial pressure following transjugular intrahepatic portosystemic stent-shunt for varicealhaemorrhage. J. Hepatol..

[38] Somberg K.A., Riegler J.L., LaBerge J.M., Doherty-Simor M.M., Bachetti P., Roberts J.P., Lake J.R. (1995). Hepatic encephalopathy after transjugular intrahepatic portosystemic shunts: Incidence and risk factors. Am. J. Gastroenterol..

[39] Rossle M., Deibert P., Haag K., Ochs A., Olschewski M., Siegerstetter V., Hauenstein K.H., Geiger R., Stiepak C., Keller W. (1997). Randomised trial of transjugular-intrahepatic-portosystemic shunt *vs*. endoscopy plus propranolol for prevention of varicealrebleeding. Lancet.

[40] Sanyal A.J., Freedman A.M., Shiffman M.L., Purdum P.P., Luketic V.A., Cheatham A.K. (1994). Portosystemic encephalopathy after transjugular intrahepatic portosystemic shunt: Results of a prospective controlled study. Hepatology.

[41] Nolte W., Wiltfang J., Schindler C., Munke H., Unterberg K., Zumhasch U., Figulla H.R., Werner G., Hartmann H., Ramadori G. (1998). Portosystemic hepatic encephalopathy after transjugular intrahepatic portosystemic shunt in patients with cirrhosis: Clinical, laboratory, psychometric, and electroencephalographic investigations. Hepatology.

[42] Mamiya Y., Kanazawa H., Kimura Y., Narahara Y., Yamate Y., Nakatsuka K., Sakamoto C. (2004). Hepatic encephalopathy after transjugular intrahepatic portosystemic shunt. Hepatol. Res..

[43] Guevara M., Baccaro M.E., Ríos J., Martin-Llahi M., Uriz J., Ruiz del Arbol L., Planas R., Monescillo A., Guarner C., Crespo J. (2010). Risk factors for hepatic encephalopathy in patients with cirrhosis and refractory ascites: Relevance of serum sodium concentration. Liver Int..

